# Crystallographic, electronic and vibrational properties of 2D silicate monolayers

**DOI:** 10.1107/S1600576725000731

**Published:** 2025-02-17

**Authors:** Gianfranco Ulian, Giovanni Valdrè

**Affiliations:** a Biological, Geological and Environmental Sciences University of Bologna P. Porta San Donato 1 Bologna Emilia-Romagna40127 Italy; b Interdisciplinary Centre of Biomineralogy, Crystallography and Biomaterials University of Bologna P. Porta San Donato 1 Bologna Emilia-Romagna40127 Italy; Australian Synchrotron, ANSTO, Australia

**Keywords:** 2D phyllosilicate, phlogopite, point defects, electronic properties, vibrational properties, density functional theory

## Abstract

Phyllosilicates are promising materials for optoelectronic applications because of their interesting electronic and magnetic properties that can be modulated by specific ionic substitutions. They can be easily exfoliated down to a single layer, enabling their use in specific 2D applications, such as the creation of van der Waals heterostructures and other materials with tailored physical properties. The present work reports a theoretical investigation of the structural, electronic, Raman and infrared properties of the (001) monolayer of phlogopite [K(Mg,Fe)_3_Si_3_AlO_10_(OH)_2_, with Mg/Fe ratio ≥ 2] and how they change with different Fe^2+^/Mg^2+^ substitutions in the structure. Although other cations could occupy the octahedral sheet positions in phlogopite (and phyllosilicate in general), here the focus is only on Fe^2+^/Mg^2+^ substitution. To this aim, density functional theory simulations were performed using the B3LYP functional, including long-range interactions in the physical treatment. The structure of the single layer of phlogopite showed a decrease of the tetrahedral rotation angle near the interlayer cations in comparison with that of the bulk mineral, which led to a tetrahedral sheet with a hexagonal pattern close to ideality, and an electronic band structure with a decreased band gap energy, down to about 3 eV. All results were discussed against the few available experimental and theoretical data in the scientific literature, finding good agreement but also further extending the knowledge of this interesting natural 2D material.

## Abstract 2

The present work reports a density functional theory investigation of the structural, electronic, Raman and infrared properties of the (001) monolayer of phlogopite [K(Mg,Fe)_3_Si_3_AlO_10_(OH)_2_, with Mg/Fe ratio ≥ 2] and how they change with different Fe^2+^/Mg^2+^ substitutions in the octahedral sheet.

## 1. Introduction

The development and applications of (opto)electronic devices require continuous research into their constitutive building blocks, for example, photodetectors; field effect transistors (FETs); complementary metal oxide semiconductor chips; photonic integrated circuits; neuromorphic computing; gas, chemical and biological sensors; and micro- and nanoelectromechanical systems (Lemme *et al.*, 2022 ▸). In this framework, miniaturization is the key process that allows us to increase the density of devices such as FETs to satisfy Moore’s law. A possible method to reduce the size of nanodevices is to replace or combine the typical active (*e.g.* semiconductors) and passive (*e.g.* gate insulators) bulk materials with 2D materials. The precursor of 2D van der Waals (vdW) materials is graphene, which was obtained from mechanical exfoliation techniques (Geim & Novoselov, 2007 ▸). These kinds of layered materials can also be easily stacked together without the concern of lattice mismatches to create heterostructures (or heterojunctions), *e.g.* graphene/MoS_2_ (Ulian *et al.*, 2021*b* ▸), MoTe_2_/SnSe_2_ (Cong *et al.*, 2022 ▸) and WSe_2_/BP (BP = black phospho­rous) (Zhang *et al.*, 2021 ▸), with tailored functions and electronic properties (Aftab *et al.*, 2022 ▸, Qin *et al.*, 2024 ▸).

However, the development of novel 2D optoelectronic components and devices must also consider passive materials, *i.e.* the gate insulator. Indeed, the technological viability of a specific device is controlled by the insulating layer and its interface with the active material (Illarionov *et al.*, 2020 ▸). Many active 2D materials have a hexagonal/trigonal symmetry, and hence it is desirable to find and use single-layered passive structures with similar crystallographic features.

In the framework of 2D materials, phyllosilicates, also called layered silicates, show promising properties for 2D applications (Frisenda *et al.*, 2020 ▸). Phyllosilicates are made of atomically flat layers composed of one or two tetrahedral sheets (T) of SiO_4_ units sharing three vertices (the basal oxygen, Ob), which are bound to an octahedral sheet (O) containing *M* = Mg, Al cations with sixfold coordination via the apical oxygen atoms (Oa) of the silica tetrahedra. One tetrahedral sheet can be connected to the octahedral sheet forming the T–O (TO, 1:1) phyllosilicate structure, or two tetrahedral sheets can sandwich the octahedral sheet to form the T–O–T (TOT, 2:1) phyllosilicate structure. These T–O or T–O–T layers are virtually infinite in the crystallographic *ab* plane and are characterized by strong covalent and ionic bonds. The layers are stacked along the *c* axis and are held together by weak long-range interactions (*e.g.* vdW). Ideally, the tetrahedral sheet should form a hexagonal mesh, but in both TO and TOT minerals the T sheet is slightly distorted because of the small lattice mismatch between the T and the O sheets, which leads to a non-perfect coupling between the two layers in the bulk minerals.

The idea that we intend to pursue and elaborate on here is that the formation of the (001) monolayer surface is accompanied by a change in the internal geometry that would bring the T sheet towards the ideal hexagonal symmetry. This is an important crystallographic feature that could be exploited, for example, in the creation of 2D nano-devices made of vdW heterostructures including phyllosilicates and other hexagonal/trigonal monolayers, because it is known that layered materials can adapt their morphology to the topography of the substrate that they are deposited onto (Xu *et al.*, 2021 ▸; Xue *et al.*, 2011 ▸). Thus, the weak coupling – also called ‘van der Waals epitaxy’ (Bitla & Chu, 2017 ▸) – between different layered materials could be enhanced by their similar crystallography, which in turn could affect the properties of the heterostructure.

In recent years, phyllosilicates have received increasing interest in the context of 2D materials and, as a consequence, several studies were devoted to their experimental and theoretical characterization and their use as building blocks in vdW heterostructures (Bitla & Chu, 2017 ▸; Dlamini *et al.*, 2021 ▸; Vasić *et al.*, 2021 ▸). Most of these investigations related to micas, *i.e.* layered silicates presenting negatively charged TOT layers due to Al^3+^/Si^4+^ substitutions in the T sheet, which are balanced by the presence of monovalent (K^+^, Na^+^) or divalent (Ca^2+^) cations in the interlayer region. These TOT + *i* structures (where *i* is the interlayer cation) present the general formula *AM*_2−3_*T*_4_O_10_*X*_2_, where *A* = K, Na, Ca is the interlayer cation; *M* = Mg, Al, Fe is the octahedral cation; *T* = Si, Al is the tetrahedral cation; and *X* = OH, F, Cl, O. A further subdivision of micas (and layered silicates in general) is into trioctahedral and dioctahedral phyllosilicates when Mg and Al, respectively, occupy the octahedral sites in the O sheet. Important members of this mineral family are dioctahedral muscovite, KAl_2_(AlSi_3_O_10_)(OH)_2_, and trioctahedral phlogopite, K(Mg, Fe)_3_AlSi_3_O_10_(OH)_2_. The crystal structure is thus given by strong covalent/ionic bonds within the TOT layers on the *ab* crystallographic plane, which in turn are stacked and held together by a mix of ionic and dispersive interactions along the [001] direction (Ventruti *et al.*, 2009 ▸). These minerals can be easily found in nature (Deer *et al.*, 1992 ▸; Icenhower & London, 1995 ▸); they are non-toxic, chemically inert, elastic and flexible and can be exfoliated down to a single layer (Alencar *et al.*, 2015 ▸; Bruno & Prencipe, 2006 ▸; Moro *et al.*, 2019 ▸). From an electronic point of view, micas are suitable as passive materials because they present a wide band gap; hence they are insulators (Cadore *et al.*, 2022 ▸; Frisenda *et al.*, 2020 ▸; Ulian & Valdrè, 2023*a* ▸), with possible applications as gate dielectrics (He *et al.*, 2011 ▸). In addition, the presence of cationic substitutions could dramatically change the electronic structure of these minerals. In contrast to the stoichiometric TOT phyllosilicates (*i.e.* talc and pyrophyllite), where the stacking is due only to vdW interactions, the interlayer cation and the negatively charged 2:1 layer in micas are also held together by Coulomb (electrostatic) forces. For example, Cadore *et al.* (2022 ▸) performed a multi-methodological experimental characterization of a natural phlogopite sample containing Fe^3+^ and Fe^2+^ in the T and O sheets, respectively, corroborated by density functional theory (DFT) simulations. The authors investigated the crystal-chemistry, vibrational fingerprint (via Raman microspectroscopy) and optical properties (reflectance measurements) of a flake (several nanometres thick), finding a direct band gap of about 3.6 eV due to the presence of substitutions of iron. Cadore *et al.* (2022 ▸) also created a vdW heterojunction made of 1L-WS_2_ deposited onto phlogopite, observing a significant enhancement of optical quality of the transition metal dichalcogenide monolayer.

Nevertheless, to the authors’ knowledge, none of the work in the scientific literature explored the crystallographic implications of creating a single layer of mica in any detail (*e.g.* from mechanical exfoliation). Also, the relationships between the crystal-chemistry and the physical, electronic and magnetic properties of the phyllosilicate monolayer need to be investigated (*e.g.* to exploit them in heterostructures grown by vdW epitaxy).

**Figure 1:**
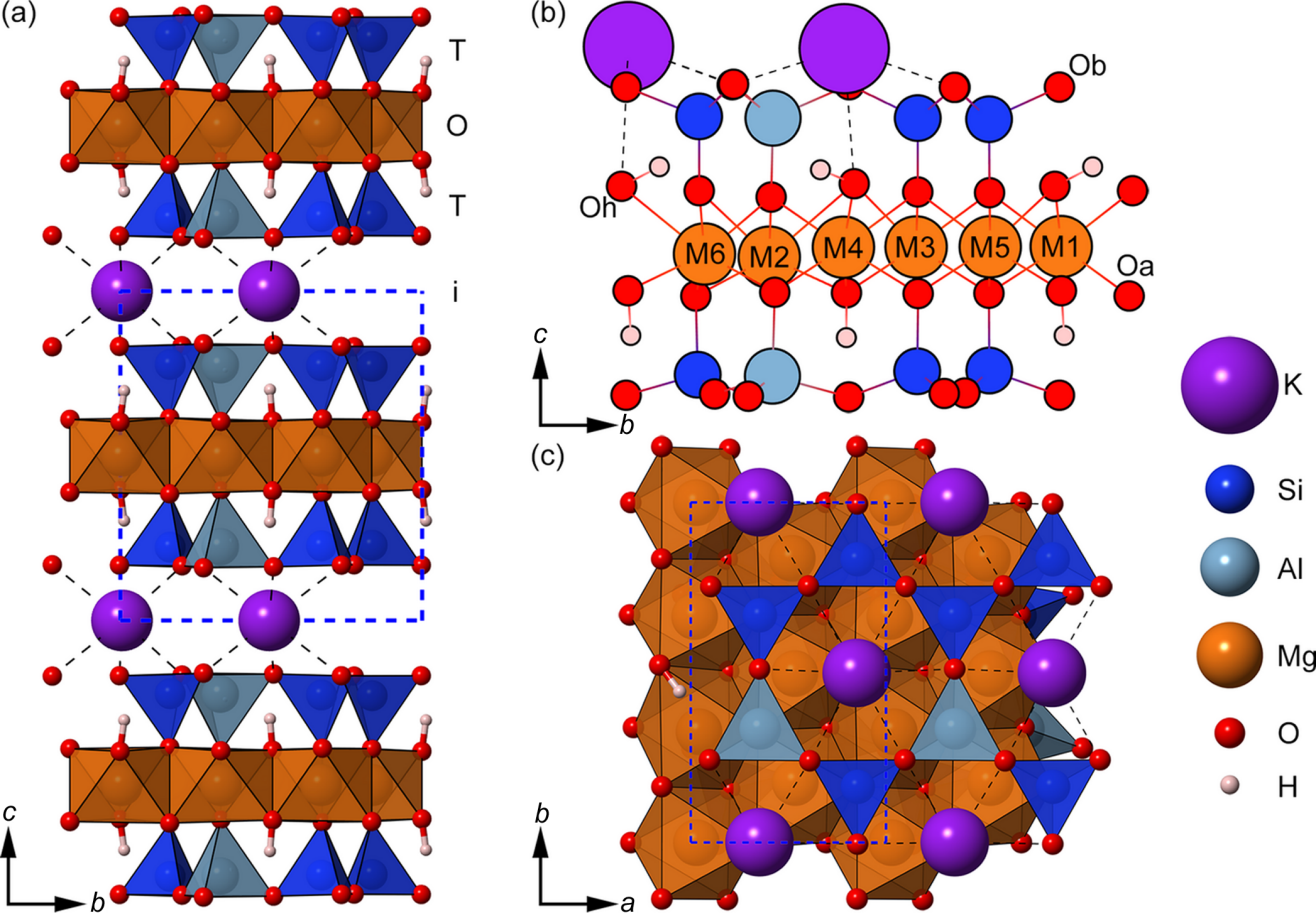
(*a*) Crystal structure of phlogopite-1*M*, represented with SiO_4_, AlO_4_ and MgO_6_ coordination polyhedra. The tetrahedral and octahedral sheets are labelled as T and O, respectively, whereas the interlayer is named *i*. (*b*) Details of the structure of an optimized TOT + *i* (001) monolayer of Phl, where the basal, apical and hydroxyl oxygen atoms are labelled Ob, Oa and Oh, respectively. The cationic sites in the O sheet are marked with the labels M1–M6. (*c*) Polyhedral representation of the (001) surface of the Phl monolayer. The blue dashed lines in the panels represent the 3D or 2D unit cell of the mineral, and the black dashed lines show the K⋯Ob interactions.

To better elucidate this idea, the focus of this work is on phlogopite, herein labelled Phl and whose crystal structure is graphically shown in Fig. 1 ▸. The (bulk) 1*M*-polytype of this mineral belongs to the space group *C*2/*m*, the monoclinic crystal system, and its lattice parameters are generally referred to the crystallographic cell [*e.g.a* = 5.3187 Å, *b* = 9.2104 Å, *c* = 10.1967 Å, α = γ = 90° and β = 100.148° (Ventruti *et al.*, 2009 ▸)]. The associated primitive cell has *a* = *b* = 5.3179 Å, *c* = 10.1967 Å, α = 84.95°, β = 95.05° and γ = 60.01°; thus, upon cleaving a single (001) layer, the resulting 2D material should ideally have *a* = *b* = 5.3179 Å and γ = 60.01° (or 119.99° if the complementary angle is considered). This means that, neglecting any possible surface reconstruction process and without considering the internal geometry, the 2D monolayer of Phl exhibits a lattice very close to the hexagonal one. However, the reconstruction process is non-negligible concerning the K^+^ position, because this cation suffers a reduction of its coordination number from 12 to 6 when the single layer is exfoliated. Indeed, as shown by the theoretical simulations of Cadore *et al.* (2022 ▸), K^+^ is drawn nearer to the basal oxygen atoms to try to restore the formal 12-fold coordination.

It is suggested for the first time here that this reconstruction process in the phlogopite monolayer is associated with a further change in the geometry of the TOT layer, in particular a decrease of the distortion of the T sheet exposed to the interlayer cation. This would mean not only that the lattice parameters can be close to those of the hexagonal crystal system but also that the tetrahedral sheet, exposed to the environment and eventually to other layered materials in van der Waals heterostructures, can have the same symmetry. This would deeply enhance the possibility of vdW epitaxy of active 2D materials onto phyllosilicates.

In addition, as shown for the bulk and monolayer phlogopite (Cadore *et al.*, 2022 ▸; Ulian & Valdrè, 2023*a* ▸), the presence of different anionic and cationic substitutions that can be found in natural samples of phlogopite and phyllosilicates, for instance Fe (Meunier *et al.*, 1983 ▸; Davidson & Yoffe, 1968 ▸), plays a relevant role in determining the electronic properties of the mineral, which must be correlated to the crystal-chemistry of the interface.

Hence, this fundamental crystal-chemical knowledge is required to design and develop future applications of 2D phyllosilicates (*e.g.* in nano-optoelectronics and magnetic materials).

The aim and novelty of the present research is to investigate in detail the intimate relationship between the crystal-chemistry and the electronic/magnetic properties of the (001) phlogopite monolayer. To this purpose, *ab initio* simulations were performed to characterize the crystal-chemical, electronic [band structure and density of states (DOS)], and Raman and infrared (IR) properties of the (001) phlogopite monolayer as a function of the iron(II) content in the TOT layer. Starting from the recently reported results on the 1*M*-polytype (Ulian & Valdrè, 2023*a* ▸,*b* ▸), a detailed DFT characterization of the above cited properties of the single layer of phlogopite was performed. The simulations were carried out (i) using a consistent theoretical approach based on the use of all-electron basis sets and the hybrid B3LYP functional for the different calculations (crystal-chemistry, electronic and vibrational properties), and (ii) considering an increasing amount of Fe^2+^ in the O sheet of the (001) phlogopite monolayers. The present systematic study shows that the exfoliation of single layers of this phyllosilicate results in a peculiar crystal structure with one tetrahedral sheet presenting a *T*O_4_ mesh close to ideality (hexagonal), a feature that is discussed here for the first time. These novel results were then discussed against the very few data reported in the scientific literature. The present results can be of interest not only for the crystal-chemical characterization of the phlogopite monolayer but also to derive important surface properties and related specific applications in various technological fields.

## 2. Computational methods

The results reported in the present work were obtained from DFT simulations carried out with the *CRYSTAL17* code (Dovesi *et al.*, 2018 ▸). As previously introduced, the chosen Hamiltonian is the well known hybrid B3LYP functional (Becke, 1993 ▸; Lee *et al.*, 1988 ▸), which includes a fraction (20%) of exact Hartree–Fock exchange and some non-local contribution to the exchange-correlation terms. The electronic energy coming from the DFT functional was corrected *a posteriori* to include the long-range interactions in the physical treatment of the mineral phases via a specifically parametrized DFT-D2 scheme for the chosen functional, a combined approach called B3LYP-D* (Grimme, 2006 ▸; Civalleri *et al.*, 2008 ▸).

Kohn–Sham orbitals were described within the linear combination of atomic orbitals (LCAO) approach, using all-electron Gaussian-type functions for each atom in the structure. In detail, K, Si, Al, Mg, Fe, O and H were described with 86-511G (Dovesi *et al.*, 1991 ▸), 88-31G* (Nada *et al.*, 1996 ▸), 85-11G* (Catti *et al.*, 1994 ▸), 8-511d1G (Valenzano *et al.*, 2007 ▸), 86-411d41G (Catti *et al.*, 1995 ▸), 8-411d11G (Valenzano *et al.*, 2007 ▸) and 3-1p1G (Gatti *et al.*, 1994 ▸) basis sets, respectively.

The reciprocal space was sampled with a 5 × 5 × 1 Monkhorst–Pack (Monkhorst & Pack, 1976 ▸) mesh with 13 independent *k* points. A pruned grid with 75 radial and 974 angular points (XLGRID) was used to calculate the total energy of the system, via numerical integration of the electron density over the unit-cell volume (Dovesi *et al.*, 2018 ▸). The self-consistent-field convergence on energy was set to 10^−8^ and 10^−10^ Ha for geometry optimization and vibrational frequency calculation, respectively. The accuracy of the Coulomb and exchange series was controlled by five thresholds set to 10^−8^ (ITOL1 to ITOL4) and 10^−16^ (ITOL5) for structural relaxation and to 10^−10^ and 10^−20^ when calculating the dielectric and polarizability tensors.

The 2D lattice and internal geometry were optimized within the same run using the analytical gradient method for the atomic positions and a numerical gradient for the slab cell parameters. The Hessian matrix is upgraded with the Broyden–Fletcher–Goldfarb–Shanno algorithm (Broyden, 1970*b* ▸,*a* ▸; Fletcher, 1970 ▸; Goldfarb, 1970 ▸; Shanno, 1970 ▸). The tolerances for the maximum allowed gradient and the maximum atomic displacement were set to 10^−5^ Ha bohr^−1^ and 4 × 10^−5^ bohr, respectively.

Zone-central (*i.e.* Γ-point) normal modes were calculated by diagonalizing the mass-weighted Hessian matrix **W** (dynamical matrix), whose elements are the second derivatives of the lattice potential for mass-weighted atomic displacements (Pascale *et al.*, 2004 ▸):
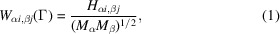
where *H*_α*i*,β*j*_ is the energy second derivative, *M*_α_ and *M*_β_ are the atomic masses, and the subscripts in Latin (*i*, *j*) and in Greek letters (α, β) are the atomic coordinates and the atoms, respectively.

The infrared spectrum was calculated analytically as a raw superposition of *p* Lorentzian functions, according to the formula
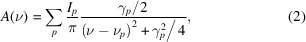
where *A*(ν) is the infrared absorbance, and ν*_p_*, γ*_p_* and *I_p_* are the vibrational frequency, the damping factor and the integrated intensity of the *p*th vibrational mode, respectively. The integrated intensity for each mode is given by
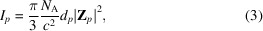
where *N*_A_ is Avogadro’s number, *c* is the speed of light, *d_p_* is the degeneracy of the mode and 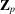 is the mass-weighted effective mode Born charge vector. The latter was obtained analytically through a coupled–perturbed Kohn–Sham approach (Ferrari *et al.*, 2009 ▸).

The Raman intensities were calculated using the approximation proposed by Placzek (1934 ▸) that considers the material as a polycrystalline powder. The intensity was then modelled with a pseudo-Voigt functional form (Maschio *et al.*, 2013 ▸):
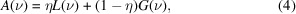
where *A*(ν) represents the Raman intensity and *L*(ν) and *G*(ν) are given by
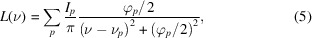

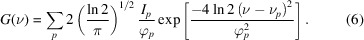
*I*_p_ is the computed Raman intensity and φ_p_ is the full width at half-maximum for the *p*th vibrational mode. η is the Lorentz factor. The typical sharp bands of Raman spectra were simulated using a pure Lorentzian form (*η =* 1). The Raman intensity of the *p*th mode was calculated according to
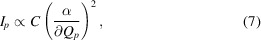
where α is the polarizability and *Q_p_* is the normal mode coordinate for mode *p*. *C* is a prefactor that depends on the (angular) frequency of the exciting laser *ω*_L_ and the temperature *T* according to
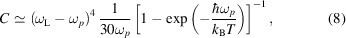
where *k*_B_ is Boltzmann’s constant and *ω_p_* is the angular frequency of mode *p*. In the simulations, the prefactor was calculated setting *T* = 298 K and ω_L_ = 532 nm to mimic possible experimental conditions. All the tensorial properties related to the intensity of the bands in the infrared and Raman spectra (*i.e.* the dielectric tensor) and the polarizability were analytically calculated using a coupled–perturbed Kohn–Sham approach (Ferrero *et al.*, 2008*a* ▸,*c* ▸,*b* ▸).

The selected atomic basis sets and computational approach were recently and successfully adopted to describe the structural, vibrational and electronic properties of bulk phlogopite (Ulian & Valdrè, 2023*a* ▸,*b* ▸) and other phyllosilicates (Prencipe *et al.*, 2009 ▸; Ulian *et al.*, 2021*c* ▸,*a* ▸, 2014 ▸; Ulian & Valdrè, 2015*a* ▸,*b* ▸).

## 3. Results

### 3.1. Crystal-chemistry of the phlogopite monolayers

The (001) phlogopite monolayer was created by virtually cutting a slab from the 1*M*-polytype of the mineral bulk, which was investigated recently by the authors of the present paper (Ulian & Valdrè, 2023*a* ▸). In the cited work, the bulk models were optimized within the space group *P*2 (monoclinic system), subgroup of *C*2/*m*, because DFT simulations cannot treat partial occupancies in the cationic/anionic sites. To cite an example, in the *C*2/*m* crystal structure obtained from neutron powder diffraction, there is a single tetrahedral site randomly occupied by *ca* 70% Si and 30% Al (Ventruti *et al.*, 2009 ▸). In quantum mechanical simulations, each site must be occupied by a single atom; hence it is not possible to directly consider structural disorder in modelling a crystalline material. The phlogopite bulk models of Ulian & Valdrè (2023*a* ▸) were in good agreement with both the experimental X-ray diffraction refinements (Ventruti *et al.*, 2009 ▸) and the theoretical results provided by Timón *et al.* (2013 ▸). In the latter work, the authors employed a similar methodology using the *CRYSTAL* code with the B3LYP hybrid functional; however they did not adopt a correction to include the contribution of long-range interactions in the physical treatment of the system.

Three (001) phlogopite monolayer structures were modelled:

(i) A stoichiometric model, with the chemical formula KMg_3_Si_3_AlO_10_(OH)_2_, labelled Phl-Fe0-ML.

(ii) A phlogopite structure presenting a single Fe^2+^/Mg^2+^ substitution occurring at the M4 site, whose chemistry is KMg_2.5_Fe_0.5_Si_3_AlO_10_(OH)_2_, named Phl-Fe1-ML.

(iii) A phlogopite model with two Fe^2+^/Mg^2+^ substitutions occurring at the M1 and M4 octahedral sites, with the chemical formula KMg_2_FeSi_3_AlO_10_(OH)_2_, labelled Phl-Fe2-ML.

A single TOT layer and the interlayer cations were extracted [shown in Fig. 1 ▸(*b*)], a procedure that does not involve breaking strong covalent bonds. Each model was geometrically optimized without any symmetry constraints (layer group *P*1), and the crystallographic results are reported in Table 1 ▸.

As expected, some reconstruction of the monolayer models occurred, especially when the interlayer cations were involved in the process. In general, the lattice parameters *a* and *b* increased by about 1.0 and 1.3%, respectively, because of the slight increase of the mean 〈*T*—O〉 and 〈M—O〉 bond distances. The only exception to this trend is represented by the K—O interactions, which instead were reduced. Considering the conversion matrix from the crystallographic to the primitive (3D) cell associated with the *C*2/*m* space group,
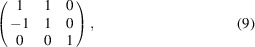
which was adapted for a layered (2D) system, the lattice parameters *a*, *b* and γ are 5.373 Å, 5.371 Å and 120.33° for the Phl-Fe0-ML model; 5.357 Å, 5.362 Å and 120.25° for the Phl-Fe1-ML system; and 5.351 Å, 5.350 Å and 120.25° for the Phl-Fe2-ML model, respectively. Thus, even without any symmetry constraints, the highest absolute deviation of the 2D lattice vectors from the ideal hexagonal symmetry is about 0.1% for the axial moduli and 0.3% for the γ angle.

**Figure 2:**
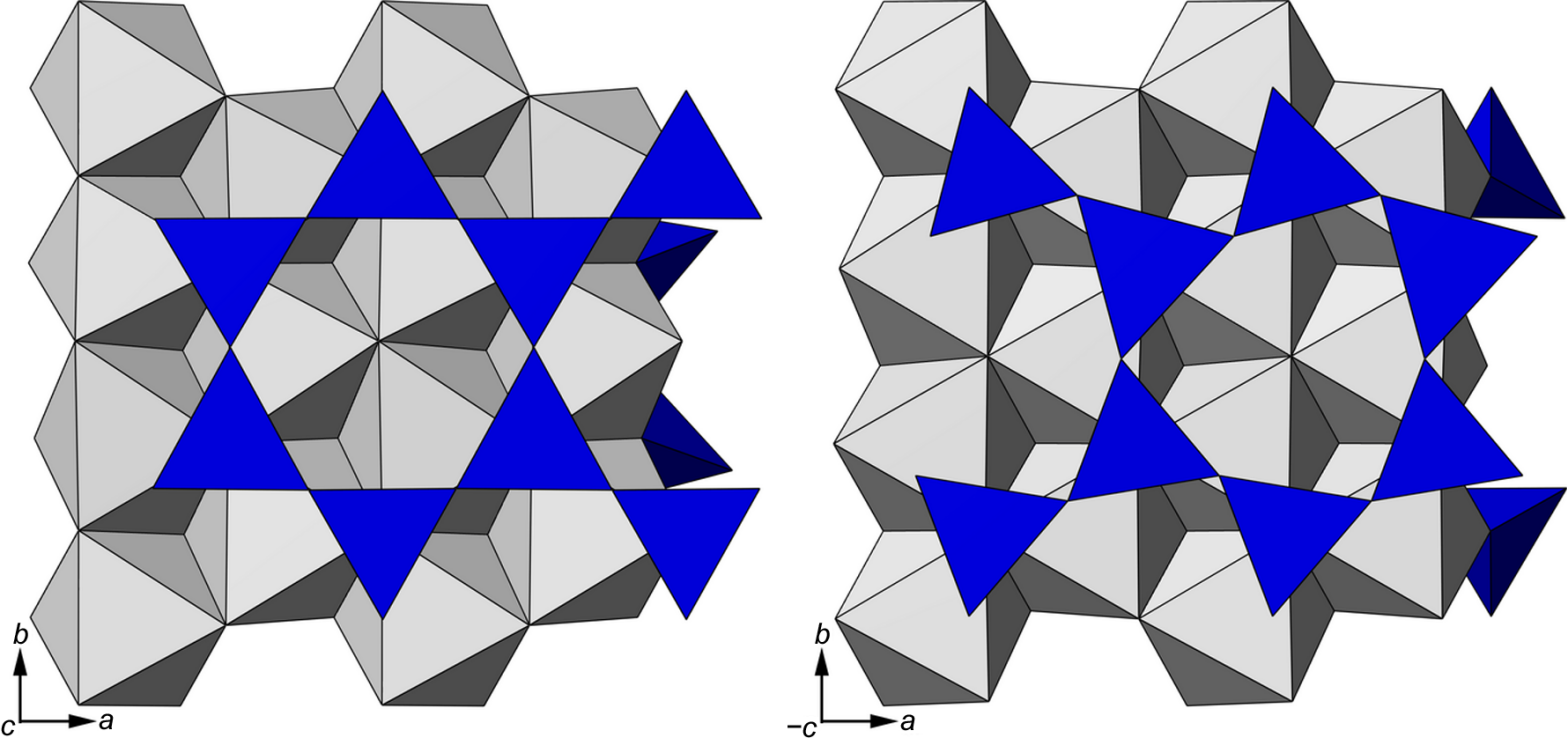
Graphical representation of the optimized structure of the Phl-Fe0-ML model, highlighting the almost ideal hexagonal upper tetrahedral sheet (on the left) and the distorted bottom T sheet.

This specific behaviour is due to the breaking of the 12-fold coordination of K^+^, which resulted in the approach of the cation towards the TOT surface, reducing the distance between the plane of the interlayer cation and the tetrahedral sheet T, *d*_K–T_, by about 50%. This movement also caused other variations in the monolayer structure, *i.e.* (i) the O—H groups canted to allow the K—Oh long-range interaction, (ii) the corrugation Δ*z* of the T sheet increased and (iii) the tetrahedral rotation angle changed significantly. The tetrahedral rotation angle α is defined as
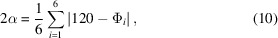
where Φ*_i_* is one of the six angles formed by the basal Ob—Ob edges of adjacent SiO_4_ tetrahedra (Valdrè *et al.*, 2009 ▸). As also shown in Fig. 2 ▸, the top T sheet of the Phl-Fe0-ML model showed an almost hexagonal (ideal) arrangement of the silica tetrahedra, with α = 0.41°, whereas the bottom T sheet was slightly more distorted (α = 12.01°) than in the mineral bulk (α = 10.76°). Similar figures were obtained also for the Fe-bearing phlogopite monolayers, proving that, in general, the side of the TOT + *i* monolayer of phlogopite exposing the interlayer cation K^+^ reaches an almost hexagonal-like structure.

The corrugation Δ*z* expresses the deviation from co-planarity of the basal Ob atoms according to
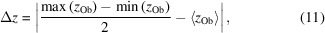
where 〈z_Ob_〉 is the mean value of the *z* coordinate of the Ob atoms in the same T sheet. Note the higher corrugation of the lower T sheet (far from K^+^ ions) than the upper one (near the interlayer cations), with differences that increase with the amount of iron in the monolayer.

The volumes of the octahedral and tetrahedral sites, *V*_M_ and *V*_T_, respectively, of the (001) Phl monolayer models are reported in Table 2 ▸. Compared with the bulk counterparts, the polyhedra are generally expanded, with the *M* sites showing a larger variation (+3.8%) than the *T* sites (+0.3%).

To check if the atomic charges were correct in the simulated models, an iterative Hirshfeld (1977 ▸) population analysis was performed. The calculated charges of K, Si, Al, Mg and Fe were +1.1, +3.2, +3.0, +2.1 and +1.9, respectively, which are in general good agreement with the formal valence states of these elements in the mineral (K^+^, Si^4+^, Al^3+^, Mg^2+^, Fe^2+^, respectively). Also, a topological analysis of the electron density was carried out using the *TOPOND* software (Gatti *et al.*, 1994 ▸), which is interfaced with the *CRYSTAL* code, to analyse the K—O interaction. According to calculated values of the electron density ρ (≃ +0.014) and its Laplacian 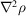 (≃ +0.056), the bond can be classified as closed-shell, which can be due to both ionic bonding and long-range interactions (Gatti, 2005 ▸). Given the calculated valence state of K, the bond is mainly ionic, with a small contribution from vdW interactions.

The surface formation energy *E*_surf_ was calculated according to the following formula:
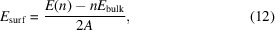
where *E*(*n*), with *n* = 1, is the energy of the monolayer structure; *E*_bulk_ is the energy of the bulk phlogopite; and *A* is the surface area. From the present theoretical simulations, the formation of the surface requires similar *E*_surf_ between the three Phl single-layer models, with values of 0.78, 0.79 and 0.80 J m^−2^ for the Phl-Fe0-ML, Phl-Fe1-ML and Phl-Fe2-ML systems, respectively. Unsurprisingly, the surface formation energy is higher than that of graphite (∼0.25 J m^−2^), MoS_2_ (∼0.21 J m^−2^) and GaSe (∼0.17 J m^−2^) (Longuinhos & Ribeiro-Soares, 2016 ▸; Ulian & Valdrè, 2023*c* ▸). This is due to the presence of ionic bonds between the TOT layers and the interlayer potassium cations, which results in a slightly more difficult exfoliation compared with typical vdW materials.

### 3.2. Electronic properties

**Figure 3:**
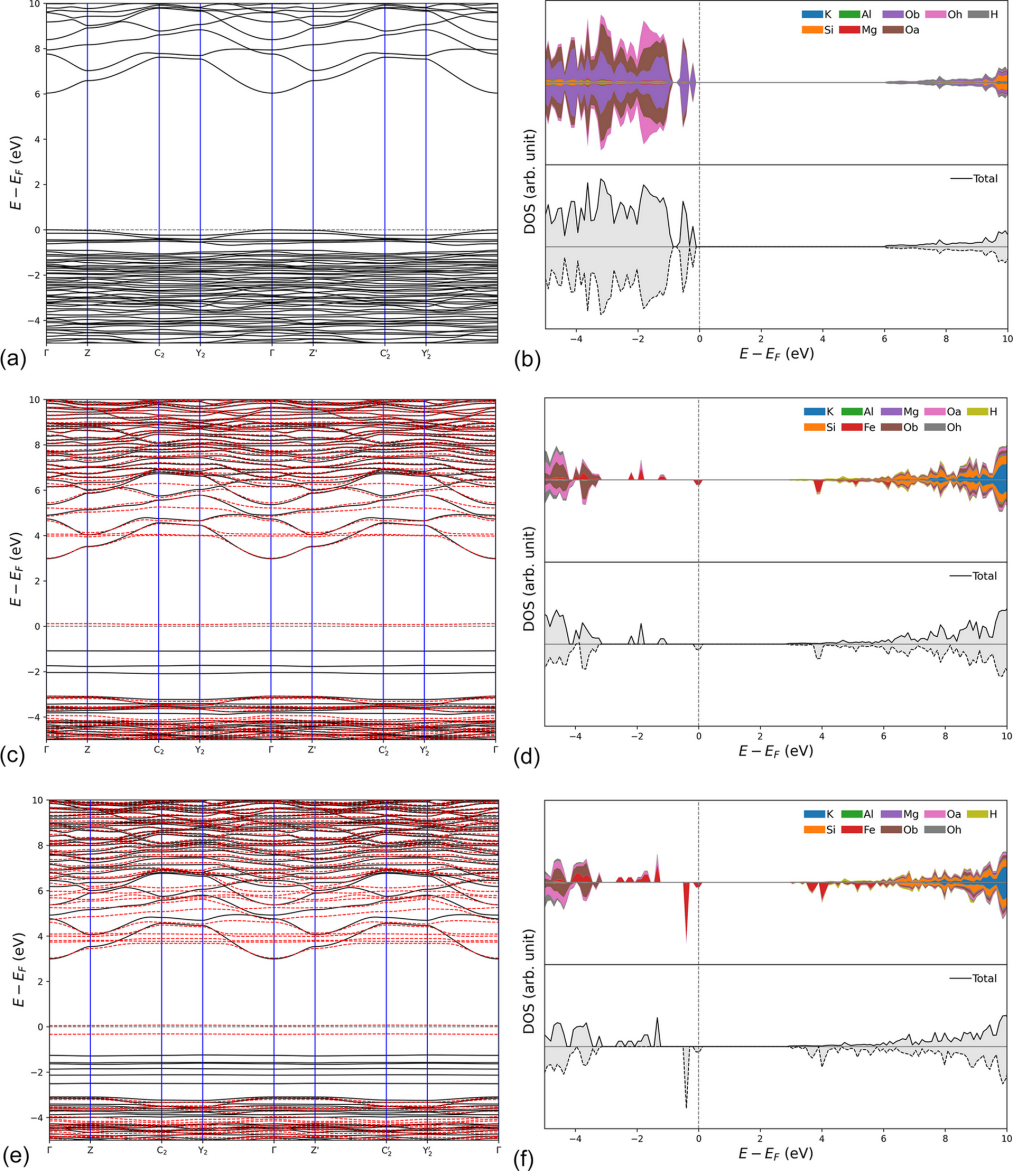
Electronic band structure and atom-projected DOS of (*a*)–(*b*) Phl-Fe0-ML, (*c*)–(*d*) Phl-Fe1-ML and (*e*)–(*f*) Phl-Fe2-ML (001) phlogopite-1M monolayer models. Black solid and red dashed lines in the band structure are associated with the α and β electrons, respectively. The atom-projected DOSs are represented with solid (positive) and dashed (negative) lines for the α and β electrons, respectively.

**Figure 4:**
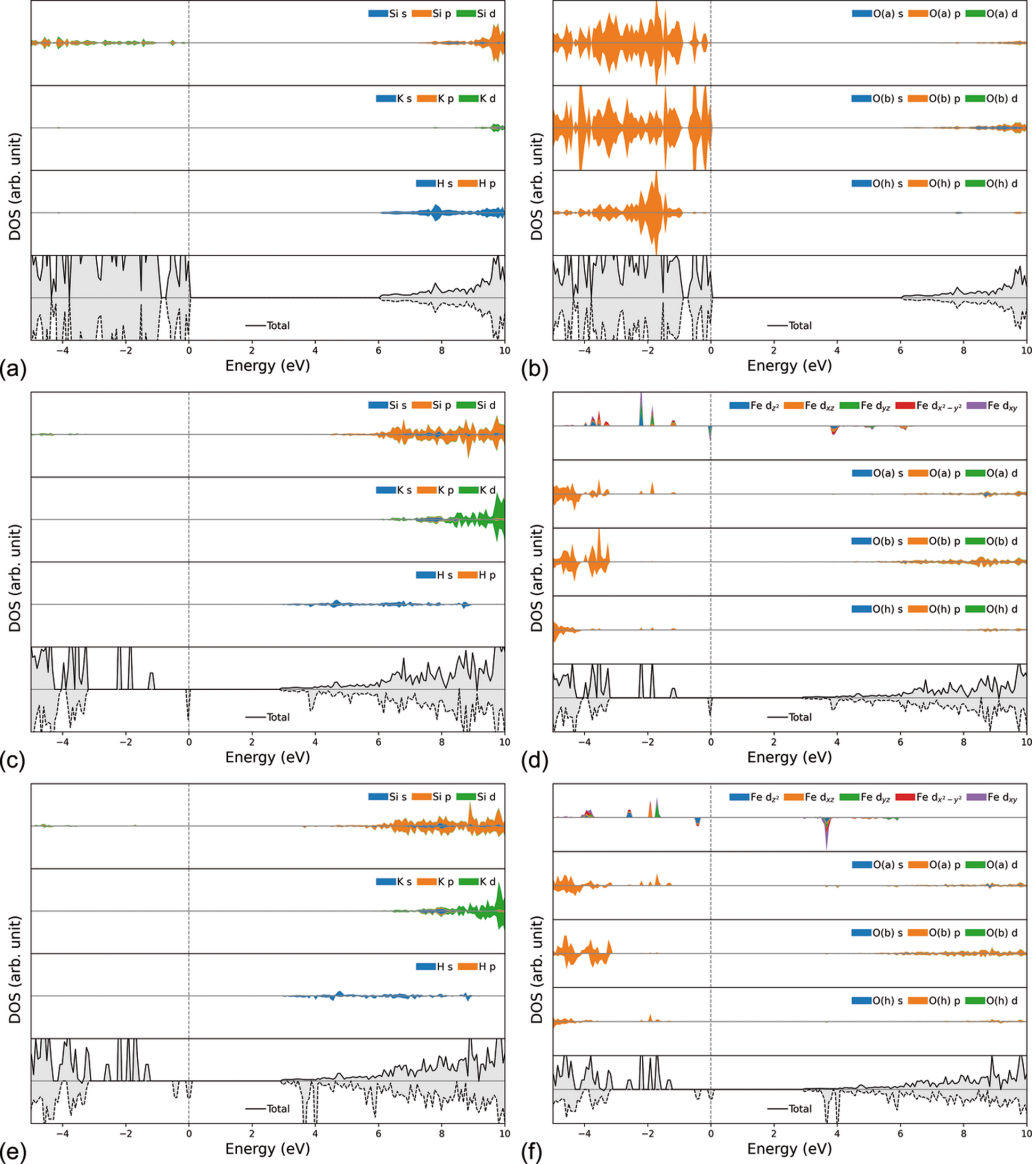
Shell-projected DOS for the different atoms in the (001) phlogopite monolayers. (*a*)–(*b*) Phl-Fe0-ML, (*c*)–(*d*) Phl-Fe1-ML and (*e*)–(*f*) Phl-Fe2-ML systems. Solid lines and dashed lines are associated with α (positive DOS values) and β electrons (negative values), respectively. The grey dashed lines show the Fermi energy level, set at 0 eV here.

The electronic band structure of the three (001) Phl monolayers was calculated in the Γ–*Z*–C_2_–*Y*_2_–Γ–*Z*′–C_2_′–*Y*_2_′–Γ path in the 2D first Brillouin zone (reciprocal 2D lattice). Because of the presence of Fe^2+^ ions having 3*d*^6^ electrons resulting in a total spin of *S* = 2, spin-polarized solutions were also calculated. Fig. 3 ▸ reports the calculated band structure for the Phl-Fe0-ML, Phl-Fe1-ML and Phl-Fe2-ML models, alongside atom-projected DOS. A more detailed analysis of the contribution of each atomic shell to the band structure is shown in the shell-projected DOS of Fig. 4 ▸. All the graphs were focused on the energy region between −5 and +10 eV.

All phlogopite monolayers presented flat valence bands near the Fermi level (0 eV), which is a behaviour commonly found in insulating materials. The band gap for the Phl-Fe0-ML model is direct in Γ, with an energy of *E*_g_ = 6.04 eV. The DOS projected on both atoms and shells showed the top valence bands are related to the *p* orbitals of Oa and Ob atoms, with very small contributions to the total DOS from the other atoms in the range −5 to 0 eV (Fermi level). In the stoichiometric phlogopite monolayer, hydrogen is responsible for the first conduction bands from about 6 eV and above. Conversely, the *p* orbitals of Si, Al and basal oxygen Ob atoms provide more states in the conduction region above about 9 eV.

The band structure of the monolayer is highly affected by divalent iron that substitutes for magnesium in the O sheet of the phlogopite monolayer [Figs. 3 ▸(*c*)–3 ▸(*f*) and Figs. 4(*c*)–4(*f*)]. Note that (i) the conduction bands were shifted at lower energies, together with most of the valence ones, (ii) Fe^2+^ bands related to the *d* orbitals appeared between those of Ob and Oa below the Fermi level, and (iii) some iron bands crossed those of hydrogen in the conduction region. Hence, the gap between the valence and conduction bands was significantly modified. For the Phl-Fe1-ML model, the computed band gap due to the spin-up electrons (α, or majority spin) was *E*_g,α_ = 4.06 eV, whereas that associated with the spin-down electrons (β, or minority spin) was *E*_g,β_ = 3.00 eV. Both gaps were direct in the Γ point in the reciprocal space. Different values were obtained for the phlogopite monolayer containing two Fe^2+^/Mg^2+^ substitutions, with *E*_g,α_ = 4.23 eV (indirect gap) and *E*_g,β_ = 3.03 eV (indirect gap). The atomic spin density calculated on the iron atoms was close to the expected value of 4, namely 3.73 μ_B_ and 3.72 μ_B_ in the Phl-Fe1-ML (single substitution) and Phl-Fe2-ML (double substitution) systems, respectively.

The analysis of the shell-projected DOS for the Fe^2+^-substituted models [Figs. 4 ▸(*c*)–4 ▸(*f*)] showed that the 2*p* orbitals of the Oa and Oh atoms were degenerate with the 3*d* orbitals (majority spin) of iron in the valence states, and that the 1*s* orbital of H was mixed with the 3*d* orbitals (minority spin) of iron in the first unoccupied electronic levels, with Fe providing the highest contribution to both the valence and the conduction bands between −2 and 4 eV. The orbitals of Ob atoms, which are not connected to any iron ion in the octahedral sheet, are only slightly involved in these degenerate levels. This result suggests that the decreased band gap in the iron-substituted phlogopite monolayer models is due to the hybridization of the Fe *d* orbitals with the *p* orbitals of Oa and Oh and the *s* orbital of H.

### 3.3. Vibrational properties of the phlogopite monolayer

One of the key techniques adopted to investigate the structure and chemistry of a material is vibrational spectroscopy (Balan *et al.*, 2001 ▸; Ulian *et al.*, 2020 ▸; Prencipe *et al.*, 2009 ▸; Wang *et al.*, 2015 ▸). For this reason, an analysis of the Raman spectrum of the phlogopite monolayer is presented in the following.

The number of degrees of freedom associated with atomic vibrational modes (phonons) are 3*N* − 3, where *N* = 44 is the number of atoms in the 2D cell. Thus, according to the *P*1 layer group of the three monolayer models, the total representation of the vibrational (optic) modes is given by
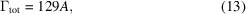
where all the modes (of *A* symmetry) are active in both infrared and Raman spectroscopies.

Since most of the spectroscopy studies on 2D materials are carried out using Raman techniques, which are useful to understand the number of layers in the sample under investigation, the focus will be on this technique.

**Figure 5:**
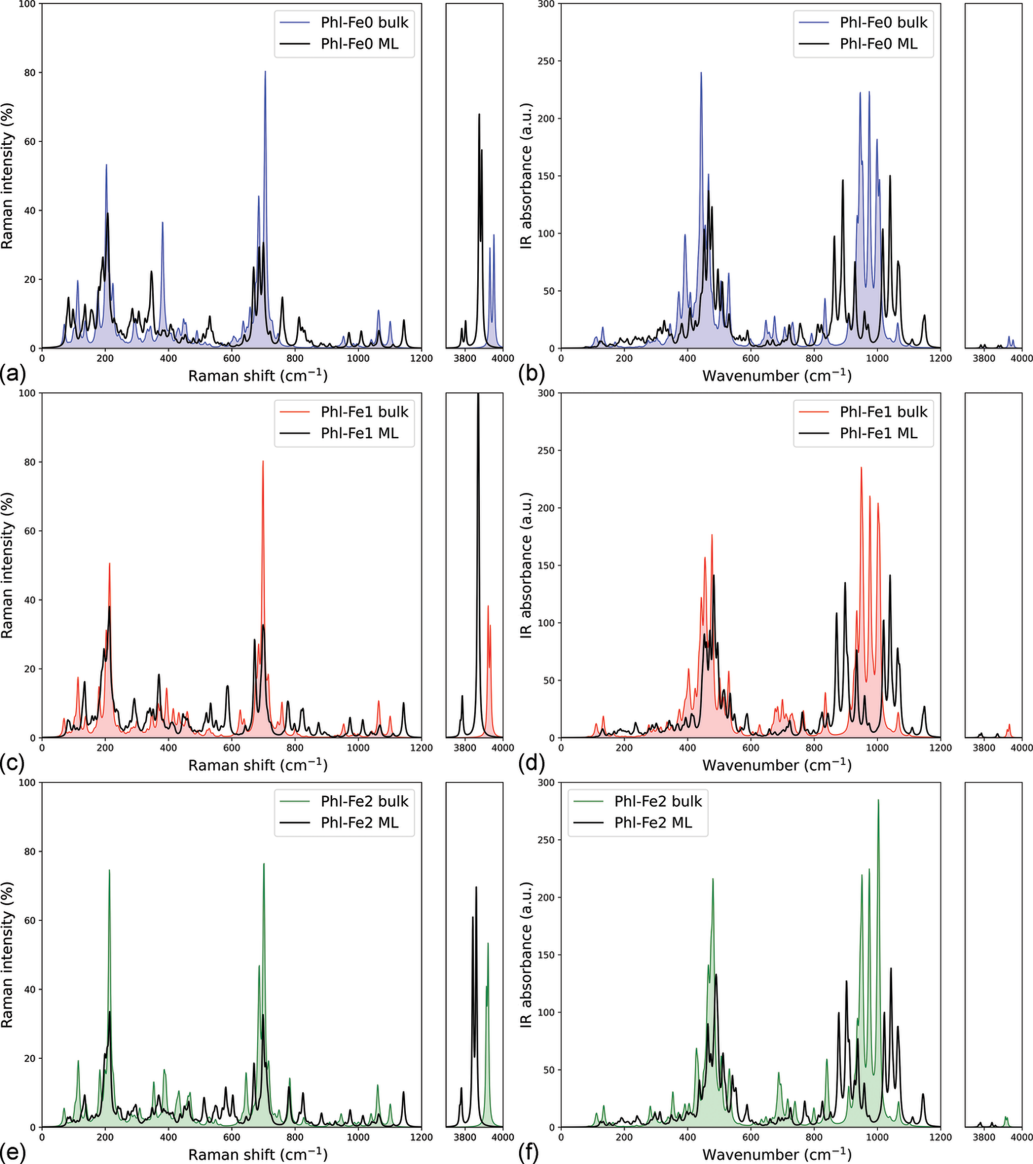
Calculated Raman spectra of (*a*) Phl-Fe0-ML, (*c*) Phl-Fe1-ML and (*e*) Phl-Fe2-ML models of (001) phlogopite monolayers, obtained for a Raman source with parallel polarization. Infrared spectra of (*b*) Phl-Fe0-ML, (*d*) Phl-Fe1-ML and (*f*) Phl-Fe2-ML structures. Results related to the 1*M*-polytype of phlogopite (bulk) are also reported for a direct comparison (Ulian & Valdrè, 2023*a* ▸).

Figs. 5 ▸(*a*), 5 ▸(*c*) and 5 ▸(*e*) report the spectra of each (001) phlogopite monolayer calculated within the harmonic approximation and using DFT, alongside the simulated spectra associated with the bulk counterparts (Ulian & Valdrè, 2023*a* ▸). The results were obtained by considering realistic experimental conditions [*i.e.* room temperature (298.15 K) and a green laser source with λ = 532 nm] and considering a parallel polarization of the incident laser with respect to the normal to the plane of the 2D mineral. The modes showing the highest variations in parallel polarization are listed in Table 3 ▸, which were assigned to specific normal modes. Also, for comparison, Table 3 ▸ reports the most intense Raman modes experimentally measured on bulk phlogopite (Ulian & Valdrè, 2023*a* ▸). Figs. 5 ▸(*b*), 5 ▸(*d*) and 5 ▸(*f*) also show the simulated infrared spectra of Phl-Fe0-ML, Phl-Fe1-ML and Phl-Fe2-ML models. Note that no atomic motion was associated with negative frequencies, *i.e.* no phonon instability occurred after the creation and relaxation of the slab models. Phonons with relatively high intensity were assigned to specific modes by analysing the potential energy distribution surface and by graphical inspection of the atomic motions using the *Moldraw* software (Ugliengo *et al.*, 1993 ▸).

In Raman spectroscopy, the phonons of the monolayers that showed the highest intensity and shift with respect to the bulk are the hydroxyl stretching modes, ν(O—H). In the phlogopite bulk, four stretching modes were present, but only two of them were visible because of the overlap of the bands (Ulian & Valdrè, 2023*a* ▸). However, in the Phl-Fe0-ML, Phl-Fe1-ML and Phl-F2-ML models, four peaks were obtained from the simulations, two of them being very intense and the other two showing weak intensity. The very strong red-shift (*i.e.* at lower wavenumbers) is due to (i) the tilting of the O—H bond from the original direction (perpendicular to the TOT plane) and (ii) the increased interaction of the Oh atom with the interlayer cation after the geometry relaxation. Hence, the strength of the O—H bond was reduced, in agreement with the longer bond distances (see Table 1 ▸), causing the observed red-shift.

More complex variations of the Raman band positions and intensities were observed in the 1000–1150 cm^−1^ range (Si—O stretching modes) and in the 600–800 cm^−1^ spectral region (O—Si—O bending modes and Mg/Fe—O—H librations). Except for the bands around 200 cm^−1^, associated with Mg/Fe—O—H librations, the other vibrational modes showed very weak intensities that could be difficult to use for the determination of the 2D phlogopite monolayer thickness.

In the infrared spectra, the most intense signals were in the 400–600 and 900–1100 cm^−1^ ranges, whereas the hydroxyl stretching region showed very weak bands. A general reduction of the intensity of the IR bands in the (001) monolayers was noted compared with the bulk phlogopite (Ulian & Valdrè, 2023*a* ▸). The most evident change when the mineral is exfoliated to a single monolayer is the red- and blue-shift of some vibrational modes in the range of the *T*—O stretching modes (*T* = Si, Al). For example, the Si—Ob stretching of the upper tetrahedral sheet falls at about 890 cm^−1^, whereas the same mode was found around 940 cm^−1^ in the bulk, with the observed red-shift in the monolayer due to the interaction of the hexagonal *T*O_4_ rings with the potassium cations. This interaction lowered the strength of the Si—Ob bonds and hence reduced the force constant of the harmonic oscillator. Conversely, the ν(Si—Ob) stretching in the lower T sheet was found at about 1040 cm^−1^, with a blue-shift of about 100 cm^−1^. The ν(Si—Oa) stretching modes that were at about 954 (medium intensity) and 975 cm^−1^ (highest intensity) were blue-shifted and red-shifted to 959 and 970 cm^−1^, respectively. The strongest band in the low-wavenumber region of the spectrum is given by the δ(O—Si—O) bending mode, falling between 470 and 490 cm^−1^ depending on the amount of iron in the octahedral sheet.

## 4. Discussion and conclusions

The data reported in the present work are relevant for the possible use of the phlogopite monolayers for optoelectronic applications. The crystallographic features of the single layers are very peculiar, showing an almost perfect hexagonal tetrahedral sheet due to the K^+^ interlayer cation approaching the TOT layer, in addition to the quasi-hexagonal 2D lattice parameters maintained even without any symmetry constraint. This is a key feature, proposed, investigated and assessed here for the first time, behind the exfoliation of this mineral. The idea of this work is to obtain and realize a specific (001) phyllosilicate surface with crystal-chemistry that can be effectively interfaced to a second layered material with hexagonal symmetry (*e.g.* graphene, hexagonal BN or transition metal dichalcogenides) to create 2D vdW heterostructures. Heterostructures, or heterojunctions, represent a way to modulate the electronic, optical and magnetic properties of 2D materials via indirect charge transfer (Mania *et al.*, 2017 ▸), plasmon–phonon coupling (Barcelos *et al.*, 2018 ▸) and other effects. In this context, the research presented here shows for the first time that the (001) phlogopite monolayer is an effective substrate for these specific applications, with electronic, magnetic and vibrational properties that can also be modulated in a controlled way by the amount of iron in the octahedral sheet of phlogopite. Note that Fe^3+^ could also be present in phyllosilicates occupying different cationic sites, *e.g.* in place of Si^4+^ in the tetrahedral sheet or in place of Mg^2+^ or Al^3+^ in trioctahedral and dioctahedral phyllosilicates, respectively. It is expected that some structural variations may occur in the presence of Fe^3+^ in the phlogopite structure, but this is beyond the scope of the present work, which focuses on the effects induced by divalent iron in the octahedral sheet of phlogopite.

The (001) phlogopite monolayer showed a remarkable band gap reduction, from about 6.94 eV in the bulk (Ulian & Valdrè, 2023*a* ▸) to 6.04 eV in the stoichiometric model, which further decreased to 3.00 eV because of the Fe^2+^/Mg^2+^ substitutions in the octahedral layer. Iron is a natural vicariant element in magnesium-bearing minerals such as trioctahedral phyllosilicates, and it is expected that the crystal-chemical and, most important, the electronic and magnetic properties will be affected by the Fe^2+^/Mg^2+^ ratio. This iron-induced modulation evaluated for the phlogopite monolayer is close to that obtained for the 1*M*-polytype of the bulk (Ulian & Valdrè, 2023*a* ▸), but the band gap was lower for the single layer.

Cadore *et al.* (2022 ▸) performed a multi-methodological experimental characterization of a natural phlogopite sample containing Fe^3+^ and Fe^2+^ in the T and O sheets, respectively. The authors investigated the crystal-chemistry, the vibrational fingerprint (via Raman microspectroscopy) and the optical properties (reflectance measurements), finding a direct band gap of about 3.6 eV. However, they were not able to properly assign the Raman fingerprint of the monolayer and few-layer phlogopite because their instrumental settings only detected the signal of the Si substrate where the phlogopite flakes were deposited (Cadore *et al.*, 2022 ▸). The authors also performed theoretical simulations to corroborate the experimental results, employing the *Quantum Espresso* DFT code with norm-conserving pseudopotentials as plane-wave basis sets to describe the atoms in the structure. The bulk and (001) monolayer of phlogopite models, stoichiometric and with a single Fe substitution in either the T or the O sheet, were geometrically optimized within the local density approximation (LDA), whereas the electronic properties (band structure and DOS) were calculated using the hybrid HSE functional (Heyd *et al.*, 2003 ▸). The 2D lattice parameters of the pristine (stoichiometric) phlogopite monolayer were *a* = 5.244 Å, *b* = 9.048 Å and γ = 90°, with the presence of a single Fe^2+^ in the octahedral sheet in place of a magnesium ion slightly increasing the cell edges (*a* ≃ 5.26 Å, *b* ≃ 9.08 Å) and reducing the angle between them (γ = 89.96°). These results are slightly underestimated compared with the present simulations carried out at the B3LYP-D* level of theory because of the well known overbinding effect of the LDA functional, *i.e.* the bond lengths and lattice parameters are generally underestimated. Cadore *et al.* (2022 ▸) calculated a band gap of about 6.2 eV for the unsubstituted phlogopite monolayer, whereas a single Fe^2+^/Mg^2+^ substitution in the octahedral sites reduced the gap to *E*_g,α_ ≃ 4.7 eV and *E*_g,α_ ≃ 3.0 eV, values that are in good agreement with those obtained from the B3LYP-D* simulations reported in the present paper. Also, the PDOS for the atoms and shells showed similar populations of the states to those shown in Figs. 3 ▸ and 4 ▸. Cadore *et al.* (2022 ▸) suggested that the band gap reduction observed when phlogopite reaches the monolayer limit is due to the stronger K—O interactions. The results provided in the present work confirm this hypothesis and extend the investigation to a double Fe^2+^/Mg^2+^ substitution. However, despite stating that phlogopite has weak vdW forces acting between the layers, the cited authors did not employ any correction to include long-range interactions within the physical treatment of the different mineral models, which are of the utmost importance for phyllosilicates, including micas (Ulian & Valdrè, 2015*a* ▸). In addition, note that the forces acting on the atoms and the unit cell were minimized with one DFT functional (LDA), but the use of another functional (HSE06) to calculate the one-electron properties (band structure and PDOS) on these relaxed structures may artificially impose a non-negligible stress state in the mineral bulk/monolayer that may bias the results. This is an important topic that is currently under investigation because the mixed functional approach has been drawing a lot of attention in the past decade. Finally, Cadore *et al.* (2022 ▸) did not provide any theoretical simulation of the vibrational properties of the bulk and (001) monolayers of phlogopite, and the effect of a double Fe^2+^/Mg^2+^ substitution on the electronic and vibrational properties was not investigated by the cited authors.

In contrast to the work of Cadore *et al.* (2022 ▸), the previous simulations of the bulk phlogopite (Ulian & Valdrè, 2023*a* ▸) and the present simulations of the (001) monolayer were carried out in a consistent manner, using a single hybrid functional (B3LYP) that was corrected with an *ad hoc*DFT-D2 scheme to include the vdW interactions in the physical treatment. Generally, hybrid functionals are better suited for the calculations of band gap and electronic properties than LDA (Ramírez-Solís, 2007 ▸; Ramos *et al.*, 2008 ▸). The results presented here show, for the first time, the formation of a hexagonal-like pattern after the surface reconstruction, thus extending the crystallographic knowledge on the phlogopite monolayer, and also are in line with the previous data reported by Cadore *et al.* (2022 ▸). The authors are aware of other computational approaches that are less empirical than the B3LYP-D* one, such as first-principles exchange-correlation DFT functionals (*e.g.* the vdW-DF family) or determinant quantum Monte Carlo methods. However, these are currently not implemented in the *CRYSTAL* code used throughout the present study. The choice of the hybrid B3LYP and a specific and calibrated DFT-D2 correction scheme for this functional (Civalleri *et al.*, 2008 ▸) was guided by the accuracy of the calculation of the crystal-chemical, vibrational and thermodynamic properties of materials and minerals, including phyllosilicates, which was proven in several previous studies by different authors (Moro *et al.*, 2019 ▸; Pascale *et al.*, 2004 ▸; Prencipe *et al.*, 2009 ▸; Ulian & Valdrè, 2015*a* ▸,*b* ▸, 2023*a* ▸,*c* ▸; Valenzano *et al.*, 2007 ▸).

The modulation of the band gap with the Mg^2+^/Fe^2+^ ratio is of the utmost importance because less energy is necessary to promote a direct transition of one electron from the top valence band to the bottom conduction band, *i.e.* an optical transition. This effect translates into the possibility of the material absorbing light with specific energy (*i.e.* wavelength) related to the *E*_g_ value according to the iron content in the octahedral sheet. The band gap is also relevant in other important applications, *e.g.* large insulators and semiconducting materials can be used as dielectric and source/drain electrodes, respectively, in field emission transistors. Furthermore, other applications could be devised from this knowledge, varying the band gap in a controlled way for the specific function of the material and/or heterojunction and, eventually, of the final device.

The presence of iron in the octahedral sheet of the phlogopite monolayer is also important for the magnetic properties. The calculated magnetic moments for the Phl-Fe1-ML and Phl-Fe2-ML monolayer models are close to those simulated for the bulk (Ulian & Valdrè, 2023*a* ▸). As also explained in the literature (Borradaile & Werner, 1994 ▸), phyllosilicates are paramagnetic at room temperature, but decreasing the temperature down to about 40 K may result in either ferromagnetic or antiferromagnetic properties depending on the number of Fe^2+^/Mg^2+^ substitutions. The magnetic character of phlogopite can then be tuned for specific applications by varying the iron content in the mineral, as well as increasing the Fe^3+^/Fe^2+^ ratio. Typically, the critical (*i.e.* Curie or Neel) temperature of phyllosilicates increases with the amount of Fe^2+^ content. Also, Fe^3+^ content may increase the ferromagnetic interactions via exchange interactions with neighbouring Fe^2+^ ions. None of these phenomena were assessed in the present work, but they will be the topic of forthcoming research.

Very few data are reported in the literature for the (001) phlogopite monolayer. The present simulations are in good agreement with previous experimental synchrotron infrared nanospectroscopy (SINS) analysis. The spectra collected by de Oliveira *et al.* (2023 ▸) on a few-layer phlogopite showed well resolved IR absorption bands centred at about 472, 508, 682, 750, 809, 851, 960, 1001 and 1062 cm^−1^. The cited authors used previous studies to assign the bands at 472 and 508 cm^−1^ to generic Si—O vibrations, and those at 960, 1001 and 1062 cm^−1^ to Si—O—Si in-plane stretchings. In our work, the theoretical analysis extends these assignments to the single-layer structure, showing that the low-wavenumber modes are associated with δ(O—Si—O) bending modes. However, de Oliveira *et al.* (2023 ▸) associated the band at 682 cm^−1^ with an Si—O—Mg mode and the three bands at 750, 809 and 851 cm^−1^ with Al—O—Si stretching modes, whereas the DFT simulations resulted in δ(Mg—O—H) bending modes (also known as OH libration) at 721, 755, 823 and 851 cm^−1^, with the latter being masked by a ν(Si—Ob) stretching mode at 864 cm^−1^ involving SiO_4_ tetrahedra near the interlayer cation. The slight deviation of the theoretical band positions from the experimental results are a consequence of several factors. The first one is that de Oliveira *et al.* (2023 ▸) carried out the SINS analysis on phlogopite flakes with thicknesses of 18 and 39 nm. In general, a single TOT + *i* layer in micas is 9.5–10.0 Å thick, which means that the thinner flake investigated by the cited authors contained about 18 layers. This suggests that the vibrational properties of the flake do not deviate significantly from that of the bulk, as also stated by de Oliveira *et al.* (2023 ▸). As a second factor, the DFT simulations were performed within the harmonic approximation, and thus the vibrational frequencies are slightly overestimated in general. A third factor is temperature, because the theoretical results were obtained at absolute zero (0 K), whereas the SINS measurements were carried out at room temperature.

Nevertheless, we have provided, for the first time, a detailed analysis of the more relevant (*i.e.* intense) infrared and Raman modes, which are useful to future experimental investigations on exfoliated phlogopite down to the single TOT + *i* layer.

As a final note, from a crystallographic point of view, the present phlogopite monolayers were modelled considering a full occupation of the K^+^ cations only on one side of the negatively charged TOT layer. The authors are aware that mechanical exfoliation may result in different figures, for instance half of the cationic sites in the ‘upper’ side of the 2:1 layer and the other half in the ‘bottom’ one. The choice made in the present work was dictated by the necessity to compare the results with previous theoretical data, and a generalization will be the subject of future work.

## Supplementary Material

CRYSTAL outputs related to the phlogopite models reported in the present paper: https://doi.org/10.17632/nrn6hfgvwn.1

## Figures and Tables

**Table 1:** Selected crystallographic features of the (001) monolayer structures of phlogopite *A* is the surface area. *T*_thick_ and *M*_thick_ are the thicknesses of the tetrahedral and octahedral sheets, respectively. α_up_ and α_down_ are the tetrahedral rotation angles of the upper and lower parts of the ML, respectively (Valdrè *et al.*, 2009 ▸). The same notation was employed to describe the corrugation Δ*z* of the basal oxygen atoms Ob of the upper and lower tetrahedral sheets of the TOT monolayer (Güven, 1971 ▸). *d*_K–T_ is the *z* distance between the plane of the interlayer cations K^+^ and the basal oxygen atoms.

	Phl-Fe0		Phl-Fe1		Phl-Fe2		Experimental
	Bulk†	(001) ML	Bulk†	(001) ML	Bulk†	(001) ML	Bulk‡
*a* (Å)	5.299	5.345	5.286	5.339	5.274	5.331	5.318–5.337
*b* (Å)	9.195	9.321	9.179	9.295	9.169	9.279	9.214–9.240
*c* (Å)	10.209	–	10.192	–	10.169	–	10.234–1.279
β (°)	100.23	90.00§	100.02	90.00§	99.80	90.00§	100.01–100.08
γ (°)	90.00§	90.02	90.00§	89.94	90.00§	90.01	90.00§
*A* (Å^2^)	–	49.83	–	49.63	–	49.46	–
*T*_thick_ (Å)	2.271	2.242	2.272	2.243	2.251	2.243	2.239–2.256
*M*_thick_ (Å)	2.178	2.212	2.162	2.196	2.144	2.176	2.140–2.200
α_up_ (°)	10.76	0.41	11.25	0.37	11.48	0.37	–
α_down_ (°)	10.76	12.01	11.25	12.45	11.48	12.64	–
Δ*z*_up_ (Å)	0.011	0.068	0.006	0.067	0.002	0.074	–
Δ*z*_down_ (Å)	0.011	0.086	0.006	0.139	0.002	0.147	–
〈Si—O〉 (Å)	1.639	1.643	1.639	1.643	1.638	1.642	1.656–1.670
〈Al—O〉 (Å)	1.752	1.759	1.752	1.758	1.750	1.757	
〈Mg—O〉 (Å)	2.077	2.112	2.074	2.114	2.073	2.119	2.073–2.090
〈Fe—O〉 (Å)	–	–	2.051	2.062	2.043	2.054	–
〈O—H〉 (Å)	0.956	0.961	0.956	0.961	0.956	0.962	–
〈K—O〉 (Å)	3.136	2.807	3.133	2.801	3.130	2.797	3.164–3.172
*d*_K–T_ (Å)	1.663	0.825	1.665	0.824	1.666	0.824	–

† Bulk results were taken from the theoretical investigation of Ulian & Valdrè (2023*a* ▸,*b* ▸).

‡ Experimental X-ray diffraction refinements (Brigatti *et al.*, 1996 ▸; Comodi *et al.*, 2004 ▸; Lacalamita *et al.*, 2012 ▸).

§ Fixed values.

**Table 2:** Polyhedral volume of *M*O_6_ (*V*_M_) and *T*O_4_ (*V*_T_) units in the (001) monolayer structures of phlogopite The tetrahedral sites labelled ‘up’ are those that face the K^+^ cations, whereas the ‘down’ tetrahedral sites are those on the opposite side of the monolayer.

	Phl-Fe0		Phl-Fe1		Phl-Fe2		Experimental
	Bulk†	ML	Bulk†	ML	Bulk†	ML	Bulk‡
*V*_M1_ (Å^3^)	11.74	11.92	11.68	11.92	11.19§	11.34§	
*V*_M2_ (Å^3^)	11.88	12.82	11.77	12.62	11.72	12.66	
*V*_M3_ (Å^3^)	11.77	12.02	11.70	11.96	11.68	11.95	
*V*_M4_ (Å^3^)	11.84	12.17	11.33§	11.52§	11.26§	11.48§	
*V*_M5_ (Å^3^)	11.71	11.95	11.70	11.96	11.61	11.89	
*V*_M6_ (Å^3^)	11.80	12.57	11.76	12.65	11.66	12.44	
〈*V*_M_〉 (Å^3^)	11.79	12.24	11.66	12.10	11.52	11.95	11.68–11.78

*V*_T1_ (up) (Å^3^)	2.249	2.259	2.245	2.253	2.246	2.248	
*V*_T1_ (down) (Å^3^)		2.244		2.243		2.242	
*V*_T2_ (up) (Å^3^)	2.252	2.295	2.248	2.288	2.247	2.281	
*V*_T2_ (down) (Å^3^)		2.262		2.262		2.257	
*V*_T3_ (up) (Å^3^)	2.267	2.281	2.264	2.274	2.261	2.266	
*V*_T3_ (down) (Å^3^)		2.248		2.244		2.243	
*V*_T4_ (up) (Å^3^)	2.757	2.773	2.752	2.765	2.748	2.753	
*V*_T4_ (down) (Å^3^)		2.750		2.747		2.744	
〈*V*_T_〉 (Å^3^)	2.372	2.380	2.368	2.375	2.366	2.370	2.33–2.36

† Bulk results were taken from the theoretical investigation of Ulian & Valdrè (2023*a* ▸,*b* ▸).

‡ Experimental X-ray diffraction refinements (Brigatti *et al.*, 1996 ▸; Comodi *et al.*, 2004 ▸; Lacalamita *et al.*, 2012 ▸).

§ Octahedral sites where Fe^2+^ substituted Mg^2+^.

**Table 3:** Selected vibrational modes of the (001) phlogopite monolayer models compared with the relative modes of the bulk mineral The numbers in parentheses are the calculated Raman intensity for the parallel polarization of the laser source, normalized to 100. In assigning the normal modes to specific vibrations, δ, l and ν indicate a bending, a libration and a stretching motion, respectively.

	Phl—Fe0		Phl—Fe1		Phl—Fe2		
Experimental	Bulk†	(001) ML	Bulk†	(001 ML	Bulk†	(001) ML	Assignment
674 (cm^−1^)	675 (13.3)	668 (10.8)	677 (14.3)	672 (32.9)	668 (2.6)	670 (22.0)	δ(O—Si—O) + l(Mg—O—H)
	685 (44.7)	686 (32.6)	685 (23.8)	691 (8.5)	687 (50.7)	687 (4.2)	δ(O—Si—O) + l(Mg—O—H)
	706 (98.1)	700 (28.8)	699 (97.6)	700 (25.2)	702 (92.2)	699 (36.8)	δ(O—Si—O) + l(Mg—O—H)

738 (cm^−1^)	746 (3.8)	760 (16.0)	759 (12.0)	778 (7.9)	749 (4.6)	770 (0.5)	l(Mg—O—H)

778 (cm^−1^)	792 (0.8)	813 (7.2)	801 (3.2)	798 (3.9)	783 (17.5)	780 (15.9)	l(Mg—O—H)

1075 (cm^−1^)	1063 (9.1)	1065 (5.3)	1064 (13.8)	1068 (2.0)	1061 (14.6)	1064 (4.0)	ν(Si—O)
	1101 (9.7)	1144 (10.1)	1101 (7.7)	1143 (12.5)	1101 (8.2)	1143 (12.7)	ν(Si—O)

3706 (cm^−1^)	3761 (1.4)	3783 (6.7)	3772 (44.2)	3775 (4.3)	3755 (1.0)	3772 (5.3)	ν(O—H), near SiO_4_
	3761 (33.9)	3803 (9.4)	3772 (0.3)	3786 (14.6)	3755 (40.1)	3781 (12.9)	ν(O—H), near SiO_4_
	3786 (39.9)	3875 (80.3)	3773 (1.1)	3868 (84.7)	3762 (60.6)	3841 (72.6)	ν(O—H), near AlO_4_
	3786 (0.4)	3888 (65.7)	3773 (35.4)	3872 (78.8)	3762 (0.3)	3859 (84.2)	ν(O—H), near AlO_4_

† Data taken from Ulian & Valdrè (2023*a* ▸).

## Data Availability

The results of the present research (simulation outputs) are freely available on a dedicated Mendeley Data repository (https://doi.org/10.17632/nrn6hfgvwn.1).
